# Refractory depression – mechanisms and efficacy of radically open dialectical behaviour therapy (RefraMED): findings of a randomised trial on benefits and harms

**DOI:** 10.1192/bjp.2019.53

**Published:** 2020-04

**Authors:** Thomas R. Lynch, Roelie J. Hempel, Ben Whalley, Sarah Byford, Rampaul Chamba, Paul Clarke, Susan Clarke, David G. Kingdon, Heather O'Mahen, Bob Remington, Sophie C. Rushbrook, James Shearer, Maggie Stanton, Michaela Swales, Alan Watkins, Ian T. Russell

**Affiliations:** 1 Emeritus Professor of Clinical Psychology, Department of Psychology, University of Southampton, UK; 2 Senior Research Fellow, Department of Psychology, University of Southampton, UK; 3 Lecturer in Psychology, Cognition Institute, School of Psychology, Plymouth University, UK; 4 Professor of Health Economics, Institute of Psychiatry, Psychology & Neuroscience, King's College London, UK; 5 Patient and Public Representative, Member of Trial Management Committee responsible for Public & Patient Inclusion, UK; 6 Professor of Social Statistics, Institute for Social and Economic Research, University of Essex, UK; 7 Visiting Professor, Consultant Clinical Psychologist, Intensive Psychological Therapies Service, Dorset Healthcare University NHS Foundation Trust, UK; 8 Professor of Mental Health Care Delivery, Department of Medicine, University of Southampton, UK; 9 Senior Lecturer in Clinical Psychology, Department of Psychology, College of Life and Environmental Sciences, University of Exeter, UK; 10 Emeritus Professor in Psychology, Department of Psychology, University of Southampton, UK; 11 Consultant Clinical Psychologist, Intensive Psychological Therapies Service, Dorset Healthcare University NHS Foundation Trust, UK; 12 Lecturer in Health Economics, Institute of Psychiatry, Psychology & Neuroscience, King's College London, UK; 13 Consultant Clinical Psychologist, Psychological Services, Southern Health NHS Foundation Trust, UK; 14 Consultant Clinical Psychologist and Reader in Clinical Psychology, School of Psychology, Bangor University, UK; 15 Associate Professor of e-Trials Research, Medical School, Swansea University, UK; 16 Professor of Clinical Trials, Medical School, Swansea University, UK

**Keywords:** Treatment-resistant depression, chronic depression, personality disorder, radically open dialectical behaviour therapy (RO DBT), randomised controlled trial

## Abstract

**Background:**

Individuals with depression often do not respond to medication or psychotherapy. Radically open dialectical behaviour therapy (RO DBT) is a new treatment targeting overcontrolled personality, common in refractory depression.

**Aims:**

To compare RO DBT plus treatment as usual (TAU) for refractory depression with TAU alone (trial registration: ISRCTN 85784627).

**Method:**

RO DBT comprised 29 therapy sessions and 27 skills classes over 6 months. Our completed randomised trial evaluated RO DBT for refractory depression over 18 months in three British secondary care centres. Of 250 adult participants, we randomised 162 (65%) to RO DBT. The primary outcome was the Hamilton Rating Scale for Depression (HRSD), assessed masked and analysed by treatment allocated.

**Results:**

After 7 months, immediately following therapy, RO DBT had significantly reduced depressive symptoms by 5.40 points on the HRSD relative to TAU (95% CI 0.94–9.85). After 12 months (primary end-point), the difference of 2.15 points on the HRSD in favour of RO DBT was not significant (95% CI –2.28 to 6.59); nor was that of 1.69 points on the HRSD at 18 months (95% CI –2.84 to 6.22). Throughout RO DBT participants reported significantly better psychological flexibility and emotional coping than controls. However, they reported eight possible serious adverse reactions compared with none in the control group.

**Conclusions:**

The RO DBT group reported significantly lower HRSD scores than the control group after 7 months, but not thereafter. The imbalance in serious adverse reactions was probably because of the controls' limited opportunities to report these.

Major depressive disorder is a recurrent, disabling condition causing substantial impairment in psychosocial functioning and quality of life.^1^ Only one-third of individuals respond fully to antidepressant medication and only half to psychological treatment.^2^ Recently treatments developed for refractory depression have achieved small-to-moderate effect sizes.^3^ Treatments are seldom effective owing to comorbidity, especially personality disorders.^4^ About half of patients with unipolar depression meet criteria for comorbid personality disorders, with higher rates among those with chronic or treatment-resistant depression.^4,5^ The commonest personality disorders among individuals with depression show excessive inhibitory control or overcontrol, including cluster A (paranoid personality disorders) and cluster C (obsessive–compulsive and avoidant personality disorders) – those that respond poorly to personality disorder treatments.^6,7^ The core characteristics of overcontrolled personality disorder are: cognitive and behavioural rigidity; strong desire to control one's environment; restrained emotional expression; limited social interaction; and problems with close relationships because of aloofness, distancing, mistrust and fear of rejection or criticism.^8^

Radically open dialectical behaviour therapy (RO DBT), a novel transdiagnostic psychotherapy, aims to address this rigid coping style.^9^ Earlier versions of RO DBT showed promise in two pilot randomised trials of patients with refractory depression and comorbid personality disorders.^10,11^ This trial aimed to assess the efficacy of RO DBT for refractory depression,^12^ and whether RO DBT causes identifiable harms.^13^

## Method

### Design

Refractory depression: mechanisms and effectiveness of radically open-dialectical behaviour therapy (RefraMED) was a three-centre parallel-group randomised trial that compared RO DBT plus treatment as usual (TAU) with TAU alone (trial registration: ISRCTN 85784627). After an internal pilot in one centre, shortage of therapists reduced recruitment below the target rate. So we extended our recruitment period from 24 months to 32; and followed the last 27 participants for 12 months (the primary end-point) rather than 18.

### Participants

Patients were eligible for the RefraMED trial if they: were 18 years or older; had an IQ more than 70; spoke English well enough to participate; had a current diagnosis of major depressive disorder according to the Structured Clinical Interview for DSM-IV-TR (SCID)-I;^14^ had refractory depression, defined as either chronic depression, that is depression lasting at least 2 years, or treatment-resistant depression, that is recurrent depression (which we operationalised as two or more previous episodes) which has not responded to an adequate dose of anti-depressant medication (ADM) for at least 6 weeks in the current episode; and had a Hamilton Rating Scale for Depression (HRSD)^15^ score of at least 15. As we had developed RO DBT specifically for overcontrol, we excluded patients who: met criteria for bipolar disorder, psychosis or dramatic-erratic personality disorders in SCID-II;^16^ had a primary diagnosis of substance dependence; or were currently receiving or waiting for standard DBT. We recruited these patients in three National Health Service (NHS) secondary care centres already delivering standard DBT for dramatic-erratic personality disorders – Dorset and Hampshire in England, and North Wales.^13^

### Interventions

#### TAU

As all three centres seek to deliver best practice, that was the natural control treatment. All participants received TAU, including prescribed antidepressant medication or psychotherapy.^13^ Participants in the control group could also access any treatment from the NHS or privately, except standard DBT. At each follow-up assessors asked participants to report their antidepressant medication and adherence to it, and psychotherapy accessed since their previous assessment or in the 6 months before their baseline assessment.

#### RO DBT

RO DBT is a transdiagnostic therapy designed to address a spectrum of disorders that are difficult to treat, notably chronic depression.^9^ It differs from other psychotherapies, notably by encouraging social bonding through emotional expression. At the time of the trial RO DBT comprised 29 weekly individual therapy sessions each lasting an hour and 27 skills training classes each lasting 2.5 h.^9,12^ The RO DBT lesson plan (supplementary Table 1 available at https://doi.org/10.1192/bjp.2019.53) included new RO DBT lessons^9^ and standard DBT lessons.^17^ RO DBT began soon after participants learned their treatment allocation. Although they continued to receive antidepressant medication as prescribed, we strongly discouraged them from seeking additional psychotherapy during RO DBT.

The RO DBT developer (T.R.L.) did not contribute to treatment delivery. He led the 10-day programme to train the 23 recruited therapists – 8 in Dorset, 10 in Hampshire and 5 in North Wales; and supervised them thereafter. Two were men, and ages ranged from 32 to 61 years. All therapists were standard DBT therapists with a minimum of 3 years clinical experience. To be recruited, therapists had to submit three treatment tapes rated as adherent on the standard DBT Adherence Coding Scale – the recognised measure of adherence in standard DBT,^18^ relevant also to RO DBT. All therapists attended weekly team meetings, to enhance treatment adherence and reduce therapist burnout. We maintained treatment fidelity across the trial by applying the standard DBT scale^18^ to randomly sampled sessions; and feeding scores back to therapists and their site leaders.

### Outcome measures

#### Primary outcome

The primary outcome was the severity of depressive symptoms 12 months after randomisation, that is 5 months after the end of treatment. Trained assessors measured this by the 17-item HRSD.^15^ Participants completed the HRSD at four points – baseline, and 7 (immediately after treatment), 12 and 18 months after randomisation. We chose 7 months rather than 6, when most clients were still attending treatment sessions, to make RefraMED more comparable with other trials that assess response to treatment immediately after that treatment. We judged it most useful to evaluate RO DBT after a full year, when remission is most important, even though psychotherapies are usually evaluated immediately after the end of therapy.

#### Secondary outcomes

We assessed remission from HRSD scores and psychosocial functioning measured by the Longitudinal Interval Follow-up Evaluation – Range of Impaired Functioning Tool (LIFE-RIFT):^19^ we defined full remission as an HRSD score below 8 and an LIFE-RIFT score below 13; and partial remission as an HRSD score below 15 and an LIFE-RIFT score below 13 points.

We measured suicidal ideation using the assessor-rated Modified Scale for Suicidal Ideation (MSSI);^20^ total scores less than nine show low ideation.

After 3 months, and the other four points, we collected data on the following potential mediating variables.

(a) Acceptance & Action Questionnaire-II (AAQ-II)^21^ measuring psychological inflexibility.
(b) Emotional Approach Coping (EAC) scale^22^ measuring emotional processing and emotional expression.
(c) Patient Health Questionnaire-9 (PHQ-9)^23^ measuring depression severity.
(d) The three-item Social Support Questionnaire (SSQ-3)^24^ measuring responders' satisfaction with support.

At baseline we also recorded potential moderating variables, notably age, gender and marital status.

### Sample size

Two pilot studies of an earlier but similar version of RO DBT for refractory depression showed effect sizes at end of treatment of 0.85^11^ and 0.71.^10^ We aimed to recruit enough participants with analysable data to yield 80% power to detect as statistically significant at the 5% level a standardised difference of 0.4 between RO DBT and TAU. We judged that clinicians and the UK National Institute for Health and Care Excellence would consider this, equivalent to a mean difference of two points on the HRSD, to be ‘clinically important’.

If there were no correlation between patients with the same therapist, a sample of 200 participants with analysable data would detect such a difference. As we aimed to collect analysable data from at least 83% of participants, we increased our target to 240. To focus on the mechanisms of RO DBT we randomised in the ratio 3 : 2 by allocating 144 ‘unclustered’ patients to RO DBT and 96 to TAU. However, participants in the RO DBT group cluster by therapist. To allow for an intratherapist correlation coefficient (ICC) of 0.025 between HRSD scores, and an average cluster size of 11 participants for each of the expected 16 therapists, we increased the RO DBT sample size to 180, yielding the same statistical power as 144 unclustered participants. Thus, we aimed to randomise 276 patients – 180 to RO DBT and 96 to TAU. We planned no interim analysis or stopping rule apart from that imposed by funding.

### Randomisation and masking

Once we had confirmed eligibility and received informed consent through the form approved by Hampshire Research Ethics Committee, we randomised participants between treatments. We used three stratifying variables to ensure balance between groups – early or late onset of depression, HRSD score above or below 25, and presence or absence of personality disorders. Within the RO DBT group, we randomised participants between available therapists so as to use as many as feasible of the treatment slots at each centre. To minimise risk of subversion, the Swansea Trials Unit used dynamic randomisation to make these allocations stochastically rather than deterministically.^25^ They emailed the resulting allocations to the trial manager for dissemination to participants and study therapists, but not assessors.

To keep assessors masked to treatment allocations they: conducted assessments away from treatment centres; asked participants not to reveal their allocations during assessments; and avoided clinical notes after initial assessment. If an allocation were revealed, we remasked by using another assessor for later assessments. If the allocation were revealed during assessment, we used the unmasked ratings; this happened 17 times at month 7, 12 times at month 12 but not at all at month 18.

### Assessor reliability

A clinical psychologist experienced in administering SCID and HSRD in clinical trials (H.O'M.) trained assessors to administer all these outcome measures. The minimum requirement for RefraMED assessors was a degree in psychology or closely related field. In reality all assessors had postgraduate qualifications, mainly MSc, DClinPsy or PhD. We discussed queries at weekly consensus meetings. We assessed interrater reliability for the HRSD at 9-month intervals across nine assessors. We analysed the reliability of individual items, more rigorous than analysing total scores. Across all measurements Krippendorff's alpha was 0.89 (95% CI from 0.86 to 0.92), implying ‘very good’ to ‘near perfect’ interrater reliability.^26^

### Statistical methods

To create a data-set for analysis, we linked study data-sets by randomisation codes. We validated this database by comparing information across sources, and by entering data twice. We scored all measures according to their published rules for imputing missing data.

We used the lmerTest package for the statistical language R to fit linear mixed-effects models to primary and secondary outcomes over the 18 months from baseline.^27^ Covariates included treatment allocated, treatment centre, baseline HRSD score, early or late onset of depression, and presence or absence of personality disorders at baseline. We used a three-level mixed-effects model to account for clustering of data by patient and therapist, avoiding the assumption that all therapists are equally effective. These mixed models are efficient and unbiased when data are missing at random. Without suitable auxiliary data we did not impute missing responses, for example by multiple imputation. However, when fewer than 10% of items were missing in a given scale, we imputed them by linear regression using the other scale items as covariates. For each outcome we estimated the main effects of treatment allocation and time, and the interaction between them; and compared groups at months 7, 12 and 18 by treatment allocated.

In assessing remission from depression, we used Button's criterion of 17.5% change in HRSD scores from baseline.^28^ We refitted our mixed models using the Bayesian software Stan, and the associated R package ‘brms’.^29^ We assessed heterogeneity in therapist performance by ICCs, and simulated prognoses for future patients on RO DBT. Analyses *post hoc* estimated posterior odds ratios^30^ for hypotheses of interest. We derived remission rates from predictions based on continuous outcomes, so did not need to test for differences in these rates directly.

### Serious adverse events

Our report to the National Institute for Health Research (NIHR) describes how we monitored serious adverse events (SAEs).^13^ The chief investigator reviewed these immediately, and reported them to the Data Monitoring & Ethics Committee (DMEC) every year, or immediately if there was suspicion of an unexpected serious adverse reaction.

### Ethical approval and conduct

Before recruiting patients, we gained approval from the Hampshire Research Ethics Committee (National Research Ethics Service reference 11/SC/0146) and the Research Governance Department of the University of Southampton, the sponsor of this trial. We asked trial participants for consent on three occasions: before telephone screening; before baseline assessment; and before randomisation.

### Patient and public inclusion

The NIHR Mental Health Research Network and ‘Involve’, the national advisory group on public engagement, helped us recruit service users – two to the Trial Steering Committee and two to the Trial Management Group. These users contributed to patient information leaflets, managing the trial and disseminating findings.

### Data availability and role of the funding source

All non-confidential data and syntax for analyses reported here are available online (https://zenodo.org/record/1442883). The Efficacy & Mechanism Evaluation Programme, funded by the Medical Research Council and administered by the NIHR, funded this trial by grant 09/150/12. NIHR monitored the trial and appointed the independent members of the Trial Steering Committee and Data Monitoring & Ethics Committee. The grant holders were responsible for: study design; collecting, analysing, and interpreting data; writing this paper; and submitting it for publication.

## Results

### Recruitment

**Fig. 1:**
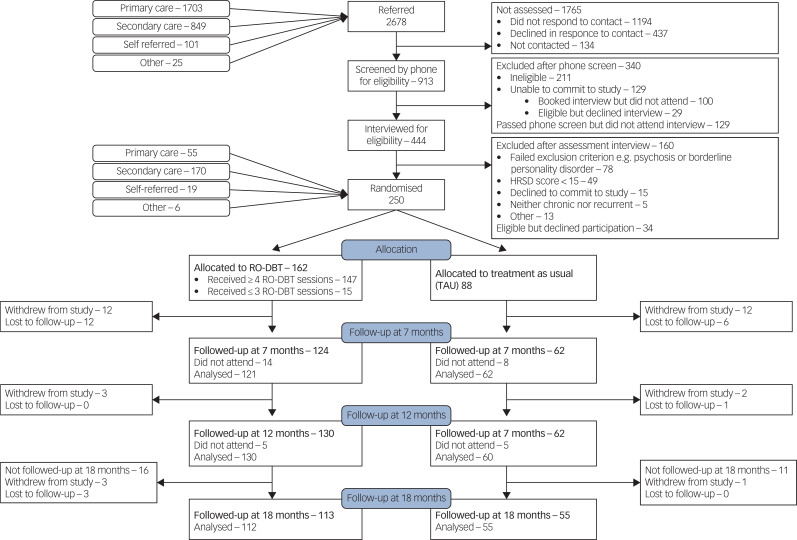
CONSORT diagram showing flow of participants through the Refractory depression: mechanisms and effectiveness of radically open-dialectical behaviour therapy (RefraMED) trial, including numbers analysed at each assessment.

Figure 1 shows the flow of participants through the RefraMED trial: we randomised 250 eligible patients, 162 (65%) to RO DBT and 88 to TAU (the control group). Recruitment started in Dorset in March 2012 with an internal pilot; started in Hampshire and North Wales in September 2012; and continued until April 2015. Of the 250 randomised participants, 170 (68%) came from secondary care, 55 (22%) from primary-care database searches, 19 (8%) from self-referral and 6 (2%) from other sources, notably private practitioners.^13^

HRSD, Hamilton Rating Scale for Depression; RO DBT, radically open dialectical behaviour therapy.

Of 162 participants allocated to the RO DBT group, 34 (21%) withdrew, including 10 who attended no sessions, 4 who attended only one or two sessions; and 10 prevented from continuing because of work or family commitments. If participants did not attend a follow-up appointment after 7 or 12 months, we asked them to attend the next scheduled follow-up. For example, 6 of the 14 RO DBT participants who did not attend their appointment after 7 months did attend their appointment after 12 months. This explains why we analysed more participants after 12 months (130) than after 7 (124).

Of the 88 participants in the control group, 22 (25%) withdrew, including 9 because they were not happy with being allocated to TAU. Only one of those withdrawing from treatment agreed to stay in the study for follow-up interviews. So the proportion of participants analysed at month 12 did not differ significantly between groups (χ^2^ = 0.71; d.f. = 1; *P* = 0.40).

### Baseline data – demographic and clinical

Of the 250 participants, 164 (66%) were women; 138 (55%) were aged between 35 and 55; 232 (97% of 238 responders) described their ethnicity as White; 106 (42%) reported being in a stable relationship; and 82 (34% of 241 responders) had a university qualification. A total of 92 participants (37%) reported a first depressive episode before the age of 16; 179 (84% of 213 responders) were chronically rather than recurrently depressed; and 191 (82% of 234 responders) had previously received psychotherapy. Our sample also showed high comorbidity: 217 (87%) with at least one Axis I disorder and 197 (79%) with at least one Axis II disorder; only 9 (4%) had no psychiatric comorbidity.^13^ Our report also confirms that our adaptive randomisation procedure was effective in balancing the characteristics of participants across groups; treatment centres were also generally comparable in terms of participants’ characteristics.^13^

### Delivery of therapy

Of 23 therapists we trained, 1 did not provide therapy within RefraMED. The number of participants seen by the other therapists ranged from 1 to 17, with a mean of 6.9 and a median of 7. The mean number of individual sessions attended by participants was 22.9 (s.d. = 6.9; 79% of the 29 planned); and the mean number of group sessions attended was 19.9 (s.d. = 7.6; 74% of the 27 planned).

### Treatment fidelity

We rated 273 (9%) tapes; and adjudged 221 (81%) of these adherent with a score of 4.0 or more.

### Primary outcome: depressive symptoms measured by HRSD

**Fig. 2:**
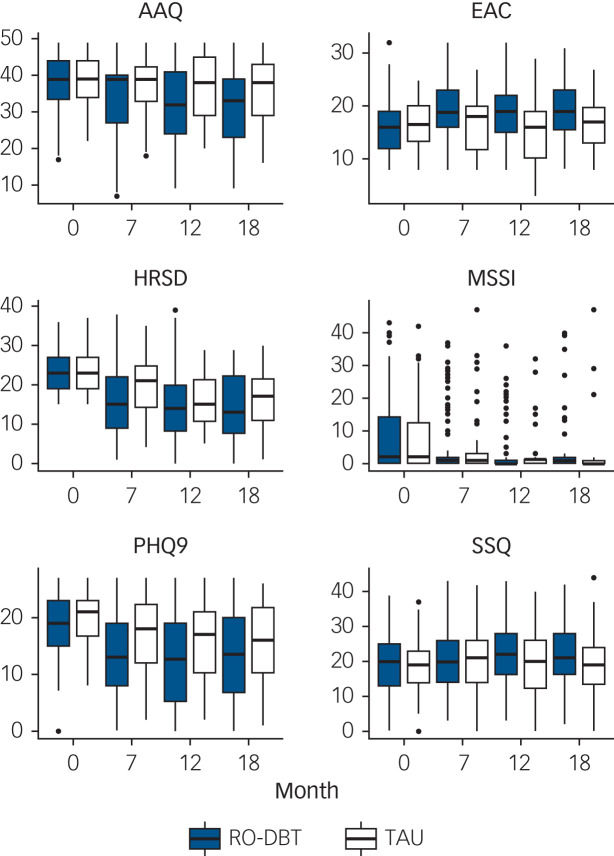
Hamilton Rating Scale for Depression (HRSD) and secondary outcomes by group.

Table 1 and Fig. 2 compare HRSD and the five secondary continuous outcome variables between groups across all follow-ups (a more detailed version of Table 1 is available in supplementary Table 2).

**Table 1:** Hamilton Rating Scale for Depression and secondary outcomes by group at 0, 7, 12 and 18 months (see supplementary Table 2 for a more detailed version of this table)

Dependent variable	RO DBT group, adjusted mean	TAU, adjusted mean	Difference	95% CI	*P*	Evidence ratio (*d* > 0)^a^
Hamilton Rating Scale for Depression						
Baseline	23.14	23.14	−0.05	−4.44 to 4.34	0.979	1.10
7 months	15.34	20.73	−5.40	−9.84 to −0.94	0.023	46.30
12 months	14.19	16.34	−2.15	−6.69 to 2.28	0.290	5.53
18 months	13.79	15.48	−1.69	−6.22 to 2.84	0.424	4.67
Action and Acceptance Questionnaire						
Baseline	38.53	38.53	0.00	−7.87 to 7.87	1.000	0.77
7 months	33.12	36.48	−3.37	−7.95 to 1.21	0.096	30.47
12 months	32.56	37.50	−4.94	−9.44 to −0.45	0.040	121.82
18 months	30.69	36.17	−5.48	−9.44 to −1.52	0.014	55.98
Emotional Approach Coping						
Baseline	16.10	16.10	0.00	−3.83 to 3.83	1.000	1.00
7 months	18.38	16.89	1.50	−0.84 to 3.83	0.132	36.36
12 months	18.64	15.10	3.55	1.22 to 5.87	0.017	3242.24
18 months	19.31	16.33	2.98	0.84 to 5.12	0.012	278.72
Modified Scale for Suicide Ideation						
Baseline	7.72	7.72	−0.00	−2.92 to 2.92	1.000	0.95
7 months	4.66	6.50	−1.84	−4.49 to 0.80	0.150	17.58
12 months	2.46	3.90	−1.44	−4.03 to 1.15	0.229	5.14
18 months	2.04	1.60	0.45	−2.48 to 3.37	0.753	1.52
Patient Health Questionnaire-9						
Baseline	19.24	19.24	−0.00	−5.02 to 5.02	1.000	0.86
7 months	13.24	16.69	−3.45	−6.61 to −0.29	0.041	30.82
12 months	13.04	15.96	−2.92	−6.04 to 0.20	0.058	33.64
18 months	12.87	16.10	−3.23	−6.05 to −0.40	0.030	12.39
Social Support Questionnaire						
Baseline	0.15	0.15	0.01	−0.44 to 0.46	0.952	0.92
7 months	0.15	−0.07	0.22	−0.23 to 0.67	0.279	0.15
12 months	0.25	−0.05	0.30	−0.15 to 0.75	0.157	0.10
18 months	0.27	−0.13	0.40	−0.08 to 0.87	0.093	0.20

RO DBT, radically open dialectical behaviour therapy; TAU, treatment as usual; OR, odds ratio.

a. Evidence ratio (*d* > 0) for hypothesis that RO DBT is better than TAU.

Box-and-whisker plots showing medians, interquartile ranges (IQRs), highest data point within 1.5 IQRs of upper quartile and lowest data point within 1.5 IQRs of lower quartile, and outliers. AAQ, Action and Acceptance Questionnaire; EAC, Emotional Approach Coping; MSSI, Modified Scale for Suicide Ideation; PHQ-9, Patient Health Questionnaire-9; SSQ, Social Support Questionnaire; RO DBT, radically open dialectical behaviour therapy; TAU, treatment as usual.

Depressive symptoms in both groups improved continuously from baseline to 18 months. By the end of therapy at seven months, RO DBT had substantially reduced depressive symptoms relative to TAU by 5.40 HRSD points (95% CI 0.94–9.85; effect size 1.03; *P* = 0.02). The RO DBT group maintained their improvement in depressive symptoms at 12 and 18 months, but the control group improved more after 7 months, reducing the difference between groups. The difference of 2.15 HRSD points at 12 months exceeded our prior target of 2 points but was not statistically significant (95% CI −2.28 to 6.59; effect size 0.41; *P* = 0.29). At 18 months the difference fell to 1.69 HRSD points (95% CI −2.84 to 6.22; effect size 0.32; *P* = 0.42). Thus, our planned contrasts revealed a statistically significant difference between RO DBT and TAU after 7 months, but not after 12 or 18 months.

**Fig. 3:**
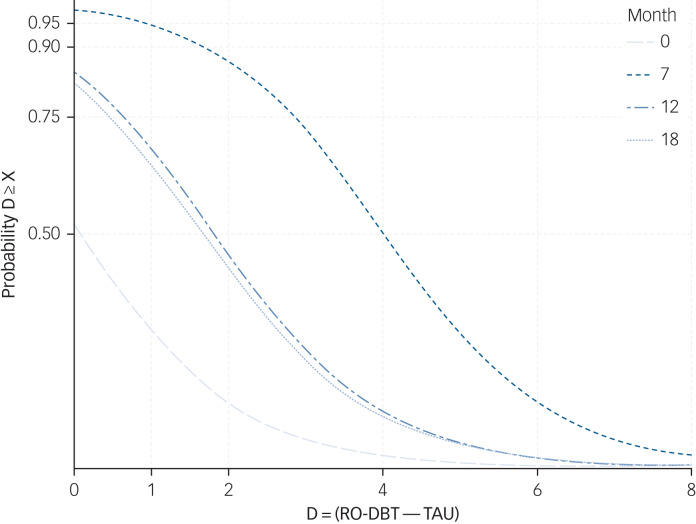
Cumulative probability that difference in Hamilton Rating Scale for Depression score between groups (*D*) exceeds X at months 0, 7, 12 and 18.

In contrast Bayesian analysis *post hoc* provided evidence that RO DBT was superior to TAU across all follow-ups: the posterior odds ratio was: 46 at 7 months – suggesting ‘strong’ evidence;^30^ and 5.5 and 4.7 at 12 and 18 months, respectively – suggesting ‘positive’ evidence.^30^ Figure 3 displays the posterior probability that RO DBT achieved the range of effects on the *x*-axis. The likely causes of the trial's reduced power was the combination of under recruitment and unexpectedly large therapist heterogeneity, yielding an ICC of 0.14, much larger than the ICC of 0.025 postulated in our power analysis. The most and least effective therapists, judged by clinical outcomes of their participants, differed by 2.6 HRSD points, equivalent to a standardised effect size of 0.43.

RO DBT, radically open dialectical behaviour therapy; TAU, treatment as usual.

### Remission rates

Using primary criteria, full remission rates were low in both groups: 1%, 8% and 7% for RO DBT and 0%, 0% and 1% in controls, at 7, 12 and 18 months, respectively; and partial remission rates were higher for the RO DBT group – 23%, 26% and 33% at successive assessments – than in control group – 6%, 22% and 24%. Using the criterion of ‘worthwhile’ change, namely 17.5% reduction in symptoms from baseline,^28^ remission rates were: 59%, 69% and 59% in the RO DBT group at 7, 12 and 18 months, respectively; and 27%, 48% and 41%, in the control group.

To help patients and clinicians interpret our findings, we simulated likely outcomes for new patients, estimating that, for every 100 new patients, 32 would achieve 17.5% improvement in symptom scores after 12 months by choosing RO DBT rather than TAU, whereas 10 would deteriorate by the same criterion, and 58 would remain essentially unchanged.

### Secondary continuous outcomes

The RO DBT group achieved significant gains in psychological flexibility and emotional coping relative to controls throughout the trial (Fig. 2, Table 1 and supplementary Table 2). Mean AAQ scores, measuring psychological inflexibility decreased over time, especially after RO DBT; the effect size increased from 0.49 (medium) after 7 months to 0.72 (large) after 12, and 0.79 after 18. EAC scores, measuring emotional coping, increased after RO DBT, but not after TAU; the effect size increased from 0.32 (small) after 7 months to 0.76 (large) after 12 months and 0.64 (also large) after 18.

However, Table 1 (and supplementary Table 2) shows no significant advantage for RO DBT in suicidal ideation or perceived social support. Mean MSSI suicidal ideation scores remained low throughout the trial for both groups; and although mean SSQ (perceived social support) scores increased after RO DBT, the difference between groups was never significant.

### SAEs

We received reports of 32 SAEs – 4 from the 88 participants allocated to TAU and 28 from the 162 participants allocated to RO DBT;^13^ none of these led to withdrawal from the trial. In the RO DBT group, 21 participants experienced a single event; 2 experienced two events each; and 1 participant experienced three events. Thus, 24 people in the RO DBT group experienced SAEs. We judged that all 4 events in the control group and 13 in the RO DBT group were ‘definitely not related’ to the study intervention, for example a leg fracture and one death from natural causes. We judged that another eight were ‘unlikely to be related’, for example recurrent non-suicidal self-injury starting before the trial. Of the remaining eight SAEs, all from the intervention group, we judged that five were ‘possibly related’ to RO DBT, including two overdoses, and three were ‘probably related’, including a prevented suicide attempt. Nevertheless, we classed none of those eight serious adverse reactions as ‘suspected unexpected’ requiring immediate reporting to the Research Ethics Committee.

Thus, all eight serious adverse reactions judged as potentially related to RO DBT occurred in the intervention group (Fisher's exact test; one-tailed *P* = 0.004). However, trial assessors saw participants in the control group only at the three follow-up interviews, so that SAE reporting relied on their volunteering relevant information. In contrast trial therapists saw the participants in the RO DBT group twice a week, and they completed diary cards reporting on suicidal ideation and self-harm. We tried to ameliorate reporting bias by asking participants' general practitioners to report any SAEs they encountered. However, no one outside the RefraMED team reported an SAE: participants in the control group reported all four SAEs either during assessment or to the trial office. In the RO DBT group therapists reported 23 (82%) SAEs, and participants reported only 5. We believe the imbalance was because of these gross differences in reporting opportunities and encouragement from therapists to use those opportunities. As in both previous trials of RO DBT,^10,11^ there were no suicides in this trial. For all these reasons the Data Monitoring Committee saw ‘no reason to suspect RO DBT had adverse effects on patients’.

## Discussion

### Principal findings

In participants with refractory depression, RO DBT was not statistically superior to TAU on the HRSD at our primary end-point of 12 months after randomisation, despite achieving the target moderate effect size of 0.40. However, it was substantially better than TAU immediately after treatment, with an effect size of 1.03, much larger than reported by previous trials of psychotherapy for refractory depression.^4^ The later fall in effect size stems from rapid improvement during RO DBT, and initially slow but accelerating improvement during TAU. Bayesian analysis *post hoc* generated ‘positive’ evidence of the superiority of RO DBT over 18 months; and suggests that 22% (i.e. 32% less 10%) more patients would experience ‘worthwhile change’ at 12 months by choosing RO DBT over TAU.

### Psychological outcomes

RO DBT aims to help individuals with rigid psychological and interpersonal styles learn flexibility. Reassuringly participants in the RO DBT group reported significantly better psychological flexibility than the controls throughout the 18 months of follow-up. RO DBT also aims to encourage appropriate expression of emotion to avoid isolation. Again, the RO DBT group reported significantly better emotional processing throughout these 18 months. Both findings suggest that participants continue to use and improve their RO DBT skills. However, there was no significant advantage for RO DBT in suicidal ideation or perceived social support. Throughout the trial suicidal ideation was low in both groups; although this decreased further over time in both groups, the difference was never significant. This finding was probably because of both groups continuing to receive treatment and support either from the trial or from the NHS. Although social support scores increased after RO DBT and decreased after TAU, the difference between groups was never significant.

### Strengths

RO DBT is the first treatment known to us to target deficits in social signalling as the main problem underlying overcontrolled emotional loneliness. We designed RefraMED as a hybrid between a phase II efficacy trial and a phase III effectiveness trial. The former yielded strengths in: the consistency of both intervention and assessment; allocating therapists to patients at random rather than allocating difficult patients to the most skilled therapists.^13^ The latter yielded strengths in: minimising exclusion criteria thus including a wide range of patients with depression and maximising generalisability; enabling the treatment developer to train therapists rather than provide therapy; and facilitating cost-effectiveness analysis.^13^ See supplementary Table 3 for the CONSORT checklist.

### Limitations

There are a range of definitions of refractory depression and treatment-resistant depression which should be taken into account when considering the generalisability of our findings. Despite our best efforts to recruit 276 participants, and analyse 229 (83%) of them, we recruited only 250 (91% of target) and analysed only 190 (76%); we also encountered unexpectedly large therapist heterogeneity. Despite achieving our target effect size at 12 months, the resulting loss of power meant our pre-planned analyses did not achieve statistical significance beyond month 7.

### Interpretation

RefraMED was the first multicentre trial of RO DBT. Although RO DBT greatly improved depressive symptoms by the end of treatment, our planned analyses were not statistically significant thereafter. Bayesian analysis *post hoc* suggests that RO DBT was superior to TAU throughout, but this effect was not clinically significant after month 7.

RO DBT does not label depression as the primary problem. Instead it targets emotional overcontrol – a maladaptive personality style known to predict the development of chronic internalising disorders such as refractory depression. Overcontrolled personality disorders, including obsessive–compulsive personality disorders, are more common than undercontrolled personality disorders; and patients' innate capacity to tolerate distress, delay gratification and avoid public displays of emotion make their problems less noticeable, and they are less likely to seek mental health treatment. Hence, it is reassuring that RO DBT improved psychological flexibility and emotional processing over 18 months in a highly symptomatic population, most of whom experience several mental health problems.

### Implications for future research

Given the recurring nature of depression, and RO DBT's aim of changing maladaptive personality, future studies should investigate long-term differences between RO DBT and other treatments. The high proportion of comorbid disorders in RefraMED (96%), and the evidence that patients with complex mental disorders do not benefit much from short-term psychotherapy,^31^ support this proposal. RO DBT's transdiagnostic approach justifies testing RO DBT across a range of conditions, including overcontrolled personality disorders (clusters A and C), anxiety disorders and eating disorders.^32^

## Supplementary material

For supplementary material accompanying this paper visit https://doi.org/10.1192/bjp.2019.53. click here to view supplementary material
